# Extract Malt and Its Combinations

**Published:** 1879-04

**Authors:** 


### Extract Malt and its Combinations.

Extracts of Malt with the preparations now made by the various Pharmaceutists seems to be rapidly taking the place of, or supplementing Cod Liver Oil and the Elixirs, formerly so extensively used. It requires time and careful observation to determine its positive value, its theoretical and apparent value is already acknowledged.

